# Biplane transrectal ultrasonography plus ultrasonic elastosonography and contrast-enhanced ultrasonography in T staging of rectal cancer

**DOI:** 10.1186/s12885-020-07369-0

**Published:** 2020-09-07

**Authors:** Yanru Feng, Chanjuan Peng, Yuan Zhu, Luying Liu

**Affiliations:** 1 Institute of Cancer Research and Basic Medicine (ICBM), Chinese Academy of Sciences, No 1, East Banshan Road, Gongshu District, Hangzhou, 310022 China; 2 Department of Radiation Oncology, Cancer Hospital of the University of Chinese Academy of Sciences, No 1, East Banshan Road, Gongshu District, Hangzhou, 310022 China; 3 Department of Radiation Oncology, Zhejiang Cancer Hospital, No 1, East Banshan Road, Gongshu District, Hangzhou, 310022 China; 4 Department of Ultrasound, Cancer Hospital of the University of Chinese Academy of Sciences, No 1, East Banshan Road, Gongshu District, Hangzhou, 310022 China; 5 Department of Ultrasound, Zhejiang Cancer Hospital, No 1, East Banshan Road, Gongshu District, Hangzhou, 310022 China

**Keywords:** Biplane transrectal ultrasonography, Ultrasonic elastosonography, Contrast-enhanced ultrasonography, Rectal cancer

## Abstract

**Background:**

The aim of this study is to assess biplane transrectal ultrasonography (TRUS) plus ultrasonic elastosonography (UE) and contrast-enhanced ultrasonography (CEUS) in T staging of rectal cancer.

**Methods:**

Between March 2016 and January 2019, 66 rectal cancer patients who completed biplane TRUS plus UE and CEUS for preoperative workup and were treated by primary total mesorectal excision (TME) were retrospectively analyzed.

**Results:**

The accuracy of TRUS plus UE and CEUS in all T staging of rectal cancer was 69.7%. The highest accuracy was achieved in the T3 stage (87.5%), while it was 71.4 and 50.0% in the T1 and T2 stage, respectively. The mean sizes of uT1-T2 lesions and uT3-T4 lesions were 30.0 ± 10.6 mm (range, 10.0–55.0) and 40.2 ± 11.2 mm (range, 14.0–57.0), respectively (*p* < 0.001). According to the receiver operating characteristic (ROC) curve to predict pT stages (pT1,2 vs. pT3), the optimal cut-off value of lesions in greatest dimension was 28.5 mm by TRUS with areas under the curve (AUC) of 0.769, and the optimal cut-off values of peak systolic velocity (PSV) and resistive index (RI) were 18.8 cm/sec and 0.645, respectively. The AUCs of PSV and RI were 0.588 and 0.555, respectively.

**Conclusions:**

Diagnostic accuracy of TRUS plus UE and CEUS in T staging of rectal cancer does not reach the excellent published study results, especially for patients with early rectal cancer. Tumor sizes, PSV and RI are useful additions for TRUS in T staging of rectal cancer.

## Background

Worldwide, colorectal cancer is the third most commonly diagnosed cancer and rectal cancer accounts for approximately one third of these cases [1]. Accurate staging is critical for rectal cancer to select appropriate therapy. In the National Comprehensive Cancer Network (NCCN) guidelines for rectal cancer, endorectal ultrasound (EUS) was suggestted when magnetic resonance imaging (MRI) was contraindicated or considered for superficial lesions [2]. Recently, a meta-analysis of comparing the diagnostic accuracy of EUS and MRI in the staging of rectal cancer indicated that EUS was superior to MRI in overall T staging [3].

In 1991, Ophir J et al. first described ultrasonic elastosonography (UE) [4]. UE is an imaging technology of strain and elastic modulus distributions in soft tissues and has been widely applicated in the liver, kidney, prostate, breast, thyroid and so on [5]. Contrast-enhanced ultrasonography (CEUS) is a technique of depicting microvessels and parenchymal perfusion with the use of specific contrast agents [6]. CEUS is complementary to ultrasonography-guided fine-needle aspiration for diagnosis, staging, and predicting treatment response [7]. However, the role of UE and CEUS for rectal cancer is limited [8–10]. The aim of this study is to assess biplane transrectal ultrasonography (TRUS) plus UE and CEUS in T staging of rectal cancer.

## Methods

### Patients

After obtaining approval from our institutional review board, rectal cancer patients (*n* = 69) who completed biplane TRUS plus UE and CEUS for preoperative workup and were treated by primary total mesorectal excision (TME) between March 2016 and January 2019 were retrospectively analyzed. Of these 69 patients, 3 were excluded from the present analysis for endoscopic submucosal dissection before TME (*n* = 1), neuroendocrine tumor (*n* = 1), and incompletion of TRUS for large tumor (*n* = 1). The present study included the remaining 66 patients. tThere were 16 female and 50 male patients with an age range of 24 to 84 years (median age, 61.5 years). The median distance from anal verge of lesion was 4.9 (0–10) cm. The median time between TRUS plus UE and CEUS testing and TME was 5 (rang, 0–40) days.

### Patient preparation prior to TRUS plus UE and CEUS

The cleaning enema was performed 1 h before examination. The Sims’ position was used and digital examination of rectum was performed to obtain the preliminary assessments of the lesion.

### TRUS plus UE and CEUS protocol

**Fig. 1:**
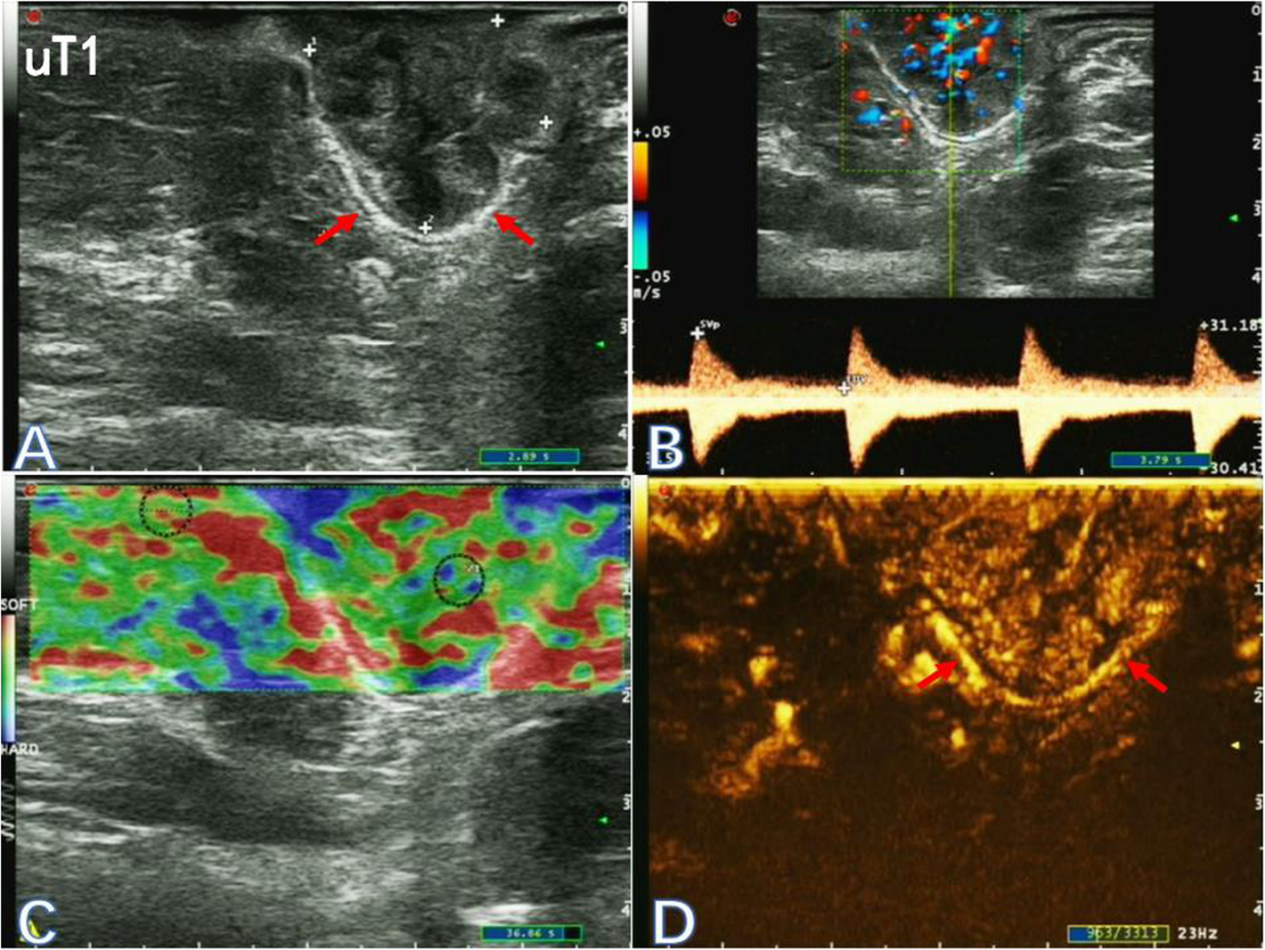
Two-dimensional ultrasonogram (**a**), color-flow and pulsed Doppler image (**b**), elastogram (**c**) and the corresponding contrast-enhanced ultrasonogram (**d**) of a 45-year-old male patient with stage uT1 low rectal cancer (red arrow)

**Fig. 2:**
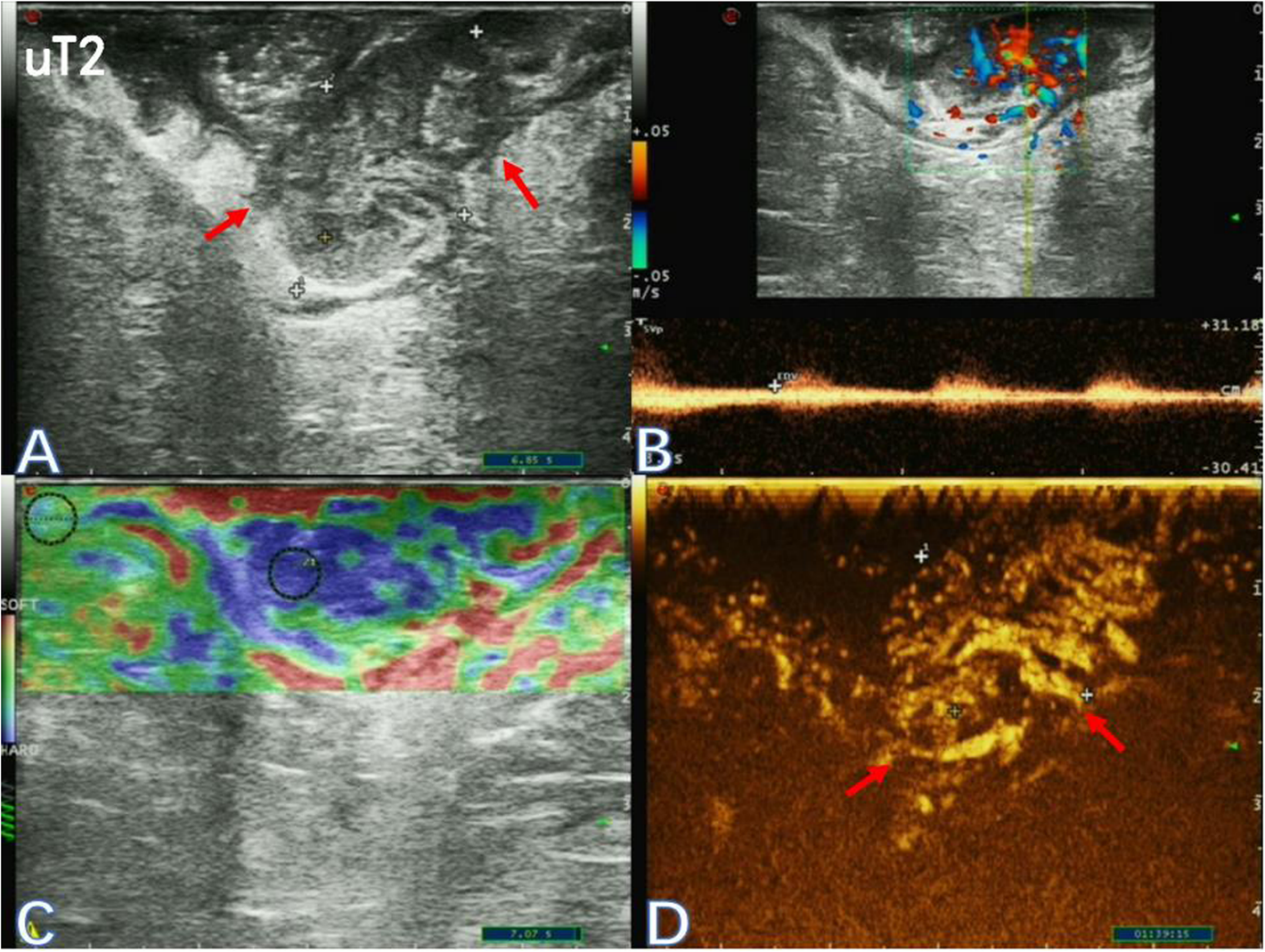
Two-dimensional ultrasonogram (**a**), color-flow and pulsed Doppler image (**b**), elastogram (**c**) and the corresponding contrast-enhanced ultrasonogram (**d**) of a 69-year-old female patient with stage uT2 low rectal cancer (red arrow)

**Fig. 3:**
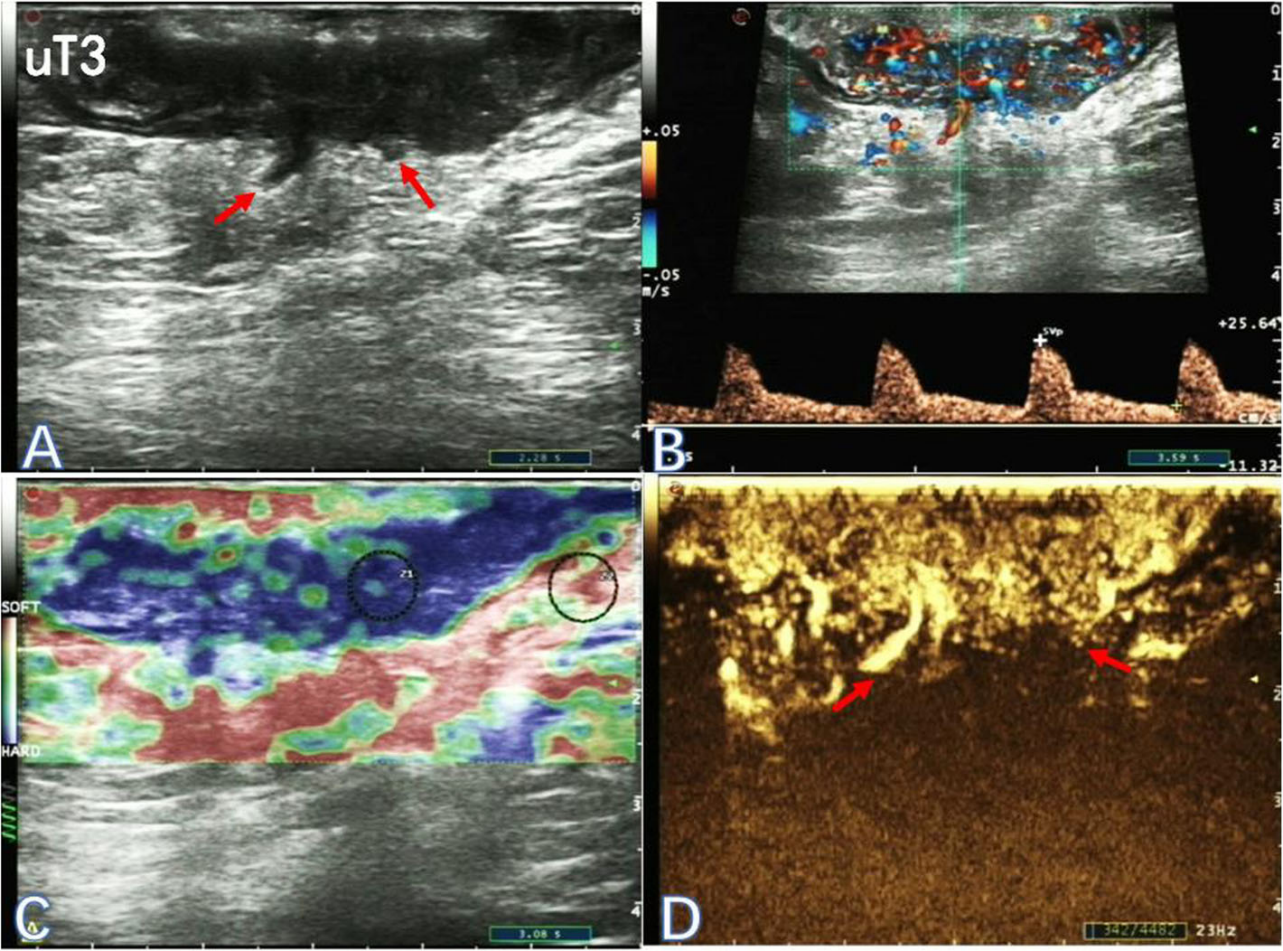
Two-dimensional ultrasonogram (**a**), color-flow and pulsed Doppler image (**b**), elastogram (**c**) and the corresponding contrast-enhanced ultrasonogram (**d**) of a 61-year-old male patient with stage uT3 low rectal cancer (red arrow)

An Ecodoppler Color Esaote MyLab™ClassC ultrasound system (Esaote, Genoa, Italy) was used, along with the TRT33 Transrectal Biplane Transducer (Esaote). Sulphur hexafluoride (SF6) lipid-coated microbubble contrast agent SonoVue™ (Bracco SpA, Milan, Italy) was used for CEUS. The methods of TRUS plus UE and CEUS were described previously [10]. Based on two-dimensional ultrasonograms, color Doppler ultrasound images, elastograms and CEUS image data, ultrasonic T classification was diagnosed for rectal cancer (Figs. 1, 2 and 3). The staging criteria by Beynon et al. [11] was adopted for ultrasonic staging and the 8th edition of the American Joint Committee on Cancer (AJCC) staging system was adopted for pathological staging. Furthemore, the primary tumor was assessed in three planes and the largest diameter was noted.

### Statistical analyses

Statistical analysis was carried out using SPSS version 22.0 (IBM, Armonk, NY, USA). Data were expressed as means ± standard deviation (SD). The predictive abilities of size of lesion, peak systolic velocity (PSV) and resistive index (RI) for pT classification (pT1,2 vs. pT3) were calculated by the receiver operating characteristic (ROC) curves. The differences between the parameters (PSV, RI and size) and pT classification (pT1,2 vs. pT3) were calculated by Chi-square and Student’s t tests. Statistical tests were based on a two-sided significance level. *p* < 0.05 indicated statistical significance.

## Results

### Biplane TRUS plus UE and CEUS in pT stages of rectal cancer

According to the 8th AJCC staging system, the pT stage distribution for all patients was 18.2% Stage pT1 (*n* = 12), 24.2% Stage pT2 (*n* = 16), and 57.6% Stage pT3 (*n* = 38). By biplane TRUS plus UE and CEUS, 7 (10.6%), 26 (39.4%), 32 (48.5%), and 1 (1.5%) patients were classified as stage uT1, stage uT2, stage uT3, and stage uT4, respectively. Of these, 46 (69.7%) patients were diagnosed with correct ultrasonic T staging. The details are shown in Tables 1 and 2.

**Table 1:** T staging of biplane TRUS plus UE and CEUS versus pathological T staging

Ultrasonic T stage	Pathological T stage (n)				Total	Ultrasonic T staging [n (%)] of patients		
	pT1	pT2	pT3	pT4		Overstaged	Understaged	Correctly staged
uT1	5	0	2	0	7	0(0.0)	2(28.6)	5(71.4)
uT2	6	13	7	0	26	6(23.1)	7(26.9)	13(50.0)
uT3	1	3	28	0	32	0(0.0)	4(12.5)	28(87.5)
uT4	0	0	1	0	1	1(100.0)	0(0.0)	0(0.0)
Total	12	16	38	0	66	7(10.6)	13(19.7)	46(69.7)

*TRUS* Transrectal ultrasonography, *UE* Ultrasonic elastosonography, *CEUS* Contrastenhanced ultrasonography

**Table 2:** Sensitivity, specificity, positive and negative predictive values for T staging of biplane TRUS plus UE and CEUS

Ultrasonic T stage	Sensitivity	Specificity	Positive predictive value	Negative predictive value
uT1	41.7%(5/12)	96.3%(52/54)	71.4%(5/7)	88.1%(52/59)
uT2	81.3%(13/16)	74.0%(37/50)	50.0%(13/26)	92.5%(37/40)
uT3	73.7%(28/38)	85.7%(24/28)	87.5%(28/32)	70.6%(24/34)
uT4	-(0/0)	98.5%(65/66)	0.0%(0/1)	100.0%(65/65)

Note, the data in parentheses represent the ratio of the number of patients

*TRUS* Transrectal ultrasonography, *UE* Ultrasonic elastosonography, *CEUS* Contrastenhanced ultrasonography

### Size of lesion

The mean sizes of uT1-T2 lesions and uT3-T4 lesions were 30.0 ± 10.6 mm (range, 10.0–55.0) and 40.2 ± 11.2 mm (range, 14.0–57.0), respectively (*p* < 0.001). According to the ROC curve to predict pT stages (pT1,2 vs. pT3), the optimal cut-off value of lesions in greatest dimension was 28.5 mm by TRUS with areas under the curve (AUCs) of 0.769. The mean sizes of pT1-T2 lesions and pT3 lesions were 33.5 ± 18.4 mm (range, 6.0–90.0) and 41.4 ± 12.2 mm (range, 13.0–80.0), respectively (*p* = 0.045). No significant difference was observed in terms of size between uT lesions and pT lesions (*p* = 0.184).

### Color-flow imaging and pulsed Doppler sonography of lesions

The mean PSV and RI were 17.5 ± 6.1 cm/sec (range, 8.3–36.0) and 0.74 ± 0.09 (range, 0.47–0.90), respectively. The mean PSV of pT1, pT2 and pT3 was 19.9 ± 7.2 cm/sec (range, 9.0–31.2), 17.4 ± 5.6 cm/sec (range, 8.3–29.3), and 16.8 ± 5.9 cm/sec (range, 9.0–36.0), respectively. The mean RI of pT1, pT2 and pT3 was 0.72 ± 0.07 (range, 0.65–0.85), 0.78 ± 0.06 (range, 0.67–0.86), and 0.73 ± 0.11 (range, 0.47–0.90), respectively. According to the ROC curve to predict pT stages (pT1,2 vs. pT3), the optimal cut-off values of PSV and RI were 18.8 cm/sec and 0.645, respectively. The AUCs of PSV and RI were 0.588 and 0.555, respectively. A marginal significant difference was observed in terms of pT stages between the PSV > 18.8 cm/sec and ≤ 18.8 cm/sec groups (*p* = 0.057). Significant difference was observed in terms of pT stages between the RI > 0.645 and ≤ 0.645 groups (*p* = 0.017).

## Discussion

The accuracy of T staging is pivotal for patients with rectal cancer in deciding on a course of therapy. The accuracy of T staging of rectal cancers by EUS has varied considerably in the literature [12]. In the present study, the accuracy of TRUS plus UE and CEUS in all T stages of rectal cancer was 69.7%. The highest accuracy was achieved in the T3 stage (87.5%), while it was 71.4 and 50.0% in the T1 and T2 stage, respectively. In the “Real World” study based on UK transanal endoscopic microsurgery database, TRUS was performed in 165 patients with uT0-T3 rectal cancer and the accuracy of TRUS in all T stages was 55.2%. The accuracy of T1, T2 and T3 lesions was 72.2% (52/72), 58.7% (27/46) and 68.8% (11/16), respectively [13]. In the prospective multicenter observational study of TRUS for local staging of rectal cancer, uT stage could be compared with pT stage in 3501 patients and the accuracy of TRUS in all T stages was 65.8%. The accuracy of T1, T2 and T3 lesions was 76.4%(307/402), 56.0% (676/1208) and 68.8% (1268/1780), respectively [14]. In another multicenter, prospective study, 7096 patients met the standards for a uT–pT comparison and the uT–pT correspondence was 64.7%. In addition, the uT-pT correspondence was higher in hospitals with a case load of over 30 per year than those with less than 10 patients per year (73.1% vs 63.2%) [15]. However, the pooled sensitivity and specificity in T staging were 79 and 89% for TRUS in the diagnostic test accuracy meta-analysis including 234 patients [3]. The different accuracy of TRUS in T staging of rectal cancer may be explained by various factors, such as the experience of the diagnostician, previous biopsy and endoscopic manipulation, peritumour inflammatory or fibrotic response, the technological developments of ultrasound and so on [13].

For the technological developments of ultrasound, the 85% accuracy was achieved in study of combining gray-scale sonography with color-flow imaging and pulsed Doppler transrectal sonography for the T staging of rectal cancer [16]. UE combined with TRUS could improve the staging of early rectal cancer [9] and CEUS was valuable for assessing microcirculation and the perfusion features of rectal cancer [8]. For patients with localized prostate cancer, multiparametric TRUS including grayscale imaging, color Doppler imaging, shear wave elastography, and contrast-enhanced ultrasound had higher sensitivity, negative predictive value, and accuracy than multiparametric MRI (97.4% versus 94.7, 96.9% versus 92.3, and 87.2% versus 76.9%, respectively) [17]. In the study including 108 patients with cervical cancer, CEUS was comparable to magnetic resonance imaging for measuring tumour size (left-right r = 0.84, craniocaudal r = 0.86 and anteroposterior r = 0.88) [18]. The 84.9% accuracy was achieved in the previous study from our center using biplane TRUS plus UE and CEUS for T staging of locally advanced rectal cancer after neoadjuvant chemoradiotherapy [10]. In the present study, the 87.5% accuracy was achieved in the T3-stage using biplane TRUS plus UE and CEUS.

Heneghan et al. reported that the mean sizes of pT1–2 and pT3–4 lesions were 2.9 ± 1.1 cm (range, 1.2–5) and 4.9 ± 2.2 cm (range, 2.6–10), respectively (*p* = 0.0016). A lesion ≥4 cm in greatest dimension could be predictive for T3-T4 by ROC curve analysis [16]. In the present study, significant difference was observed in terms of the mean sizes between uT1-T2 lesions and uT3-T4 lesions. For improving the staging of TRUS, a lesion 28.5 mm in greatest dimension by TRUS could be predictive for pT3 by ROC curve analysis. Although tumor size has not been adopted for staging of rectal cancer to date, it is helpful in uncertainty for the depth of invasion during TRUS.

RI was decreased with the increase of pT staging and PSV was significantly increased with the increase of pT staging in the study including 56 rectal cancer patients receiving TRUS [19]. In Heneghan et al’s study, significant difference was observed in terms of mean PSV between T1-T2 lesions (19.3 ± 9.2 cm/sec) and T3-T4 lesions (31.5 ± 16.3 cm/sec) (*p* = 0.048). No significant difference was observed in terms of mean RI between T1-T2 lesions and T3-T4 lesions (*p* = 0.15) [16]. With respect to the results of PSV and RI in the present study, the association of PSV, RI and T staging should be evaluated in larger cohorts from muti-centre in the future.

There are several limitations in the current study, including the retrospective nature of the study design, a single center experience, and the limited number of patients, which could affect the outcomes. Nevertheless, our report is noteworthy because this is the first study to evaluate biplane TRUS plus UE and CEUS in T staging of rectal cancer after primary TME. Ultrasonography based radiomics has been used to improve prediction of lymph node metastasis of rectal cancer [20]. The role of UE and CEUS based radiomics for rectal cancer should be elucidated in the future.

## Conclusions

Diagnostic accuracy of TRUS plus UE and CEUS in T staging of rectal cancer does not reach the excellent published study results, especially for patients with early rectal cancer. Tumor sizes, PSV and RI are useful additions for TRUS in T staging of rectal cancer.

## Data Availability

Our data can not be made publicly available for ethical reasons. Data are from the present study whose authors may be contacted at liuly@zjcc.org.cn or Department of Radiation Oncology, Zhejiang Cancer Hospital, Hangzhou, China.

## Abbreviations

TRUS: Transrectal ultrasonography
UE: Ultrasonic elastosonography
CEUS: Contrast-enhanced ultrasonography
TME: Total mesorectal excision
ROC: Receiver operating characteristic
AUC: Areas under the curve
PSV: Peak systolic velocity
RI: Resistive index
AJCC: American Joint Committee on Cancer
